# Efficacy of graphene quantum dot-hyaluronic acid nanocomposites containing quinoline for target therapy against cancer cells

**DOI:** 10.1038/s41598-024-81604-7

**Published:** 2025-03-12

**Authors:** Mozhgan Soltani, Negar Ahmadzadeh, Sarah Rajabi, Nazanin Besharati, Niloufar Khatamian, Masoud Homayouni Tabrizi

**Affiliations:** 1 Department of Biology, Mashhad Branch, Islamic Azad University, Mashhad, Iran; 2 Department of Tissue Engineering, School of Advanced Technologies in Medicine, Royan Institute, Tehran, Iran; 3 Department of Cell Engineering, Cell Science Research Center, Royan Institute for Stem Cell Biology and Technology, ACECR, Tehran, Iran

**Keywords:** Graphene quantum dots, Hyaluronic acid, Quinoline, Cancer cells, Cytotoxicity, Biochemistry, Biotechnology

## Abstract

The study aims to assess the impact of graphene quantum dot-hyaluronic acid-quinoline nanocomposites (GQD-HA-Qu NCs) on MCF-7, HT-29, A2780, PANC-1, and HeLa cell lines. The GQD-HA-Qu NCs were characterized using dynamic light scattering (DLS), field emission scanning electron microscopy (FESEM), and Fourier-transform infrared (FTIR) spectroscopy. MTT assays and flow cytometry evaluated the cytotoxic and apoptotic effects of synthesized NCs. Additionally, real-time PCR was utilized to assess apoptotic gene expression. The DLS assay revealed a particle size of 224.96 nm with a polydispersity index (PDI) of 0.3. The FESEM analysis also confirmed the uniform spherical morphology of NCs. The MTT assessment demonstrated significant cytotoxicity in all cell lines, with MCF-7 and A2780 exhibiting pronounced sensitivity (*P* < 0.001). The flow cytometry analyses also revealed a dose-dependent increase in late apoptosis at higher concentrations of GQD-HA-Qu NCs. Notably, p53 expression was significantly upregulated compared to the untreated cells (*P* < 0.01), while caspases 8 and 9 showed no substantial change. This finding indicates that the p53 pathway is predominant in mediating GQD-HA-Qu NCs-induced apoptosis. The present study suggests that GQD-HA-Qu NCs are a promising treatment with selective cytotoxicity against cancer cells and robust antioxidant activity. These findings warrant further investigation for potential clinical applications.

## Introduction

Over time, cancer treatment shifted toward targeted therapy. This evolution has resulted in drug delivery systems (DDSs) that enhance cancer treatment by targeting cancer pathophysiology^1^. In this context, nanocomposites (NCs) have emerged as a critical component of DDSs, facilitating therapeutic efficacy while minimizing adverse effects. Their distinctive characteristics, including a high surface area, biocompatibility, and controlled release capabilities, enable targeted delivery of anti-cancer agents directly to tumor sites^2^. Among the various DDSs, graphene quantum dot-hyaluronic acid (GQD-HA) nanocarriers, composed of conjugated GQDs and HA, are distinguished by their biocompatibility and capacity to facilitate controlled drug release^3,4^. The unique combination of GQDs and HA significantly improves the efficacy of GQDs in drug delivery^3^. GQDs are nanoscale carbon-based particles belonging to the graphene family. They are typically characterized by a diameter of less than 20 nm. The GQDs’ medical applications include drug delivery, biosensing, imaging, and diagnostics^5^. The minimal toxicity and unique optical and electronic properties make GQD suitable for targeting cancer cells^6^. These NCs have a high surface area, allowing efficient drug loading. In this regard, Ghanbari et al. (2024) developed a targeted DDS using GQDs using GQD that contain covalently attached folic acid and non-covalently bound tamoxifen. This system exhibited pH-sensitive release rates of 85% at pH 5.5 and 58% at pH 7.4 for 120 h. MTT assays showed low toxicity of the nanocarriers, while TMX/FA-GQDs demonstrated enhanced toxicity against MCF-7 cancer cells^7^. Moreover, their photoluminescence capability enables real-time tracking of drug delivery^4^. On the other hand, the HA component, as a natural polysaccharide, improves the drug-carrying capacity of GQD by targeting CD44 receptors, which are overexpressed in many cancer cells^8–10^. This selectivity enhances the drug’s uptake by cancer cells and reduces the side effects on healthy tissues. For example, Asghari and Mahmoudifard (2023) used HA-functionalized GQDs to detect cancer cells through changes in photoluminescence intensity, relying on the CD44 receptor-HA interaction. Results showed a reduction in GQD-HA fluorescence as the number of captured cancer cells increased, highlighting the system’s selectivity and specificity for accurate cancer diagnosis using fluorescent imaging^11^.

In addition, HA prevents aggregation and helps maintain the integrity of NCs in physiological environments^12,13^. The biocompatibility and biodegradability of HA reduce the likelihood of immune responses, while its moisture-retaining capacity makes it suitable for applications in tissue engineering^12^. The physicochemical properties of GQDs facilitate efficient drug loading and release, while the HA component ensures selective targeting of cancer cells. Consequently, the optical and electrical properties of GQD-HA make them appropriate nanocarriers for targeted therapy in cancer^14^. Moreover, the high surface area of these NCs allows efficient drug loading, while their photoluminescence enables real-time drug delivery tracking^15,16^. GQD-HA can also improve the solubility and stability of medicines, thereby enhancing their bioavailability^9^. Furthermore, their ability to generate intracellular reactive oxygen species (ROS) may enhance their effectiveness by triggering apoptosis in cancer cells^17,18^. Given the promising properties of GQD-HA NCs in enhancing drug delivery, incorporating quinoline as a therapeutic agent could further optimize their efficacy against cancer.

In this regard, quinoline (C9H7N) is a suitable candidate for delivery to cancer cells via NC carriers^19^. Quinoline is a heterocyclic aromatic organic compound with a double-ring structure composed of benzene and pyridine rings. It is a versatile scaffold for drug discovery, providing a structural framework for developing various bioactive compounds with diverse pharmacological properties^20^. Quinoline and its derivatives exhibit anti-cancer capacity by modulating biological pathways involved in cancer progression. It can induce apoptosis, inhibit cell proliferation, and interfere with tumor growth signaling pathways^21,22^. Based on this evidence, we hypothesized that conjugation with GQD-HA NCs would enhance quinoline bioavailability, optimize the dosage, and improve cancer cell targeting. As a result, this study was conducted to investigate the cytotoxicity efficacy of these NCs against some cancer cell lines, including MCF-7 (human breast cancer), HT-29 (human colorectal adenocarcinoma), A2780 (human ovarian adenocarcinoma), PANC-1 (human pancreatic carcinoma), and HeLa (human cervical carcinoma). This study introduces an innovative approach by utilizing GQD conjugated with hyaluronic acid (HA) as a nanocarrier for targeted delivery of quinoline to cancer cells, thereby enhancing the drug’s bioavailability. The comprehensive characterization and analysis of GQD-HA NCs provide valuable insights into their mechanisms, paving the way for future clinical applications in cancer therapy.

## Materials and methods

### **Fabrication of GQD-HA-Qu**

The fabrication of GQD-HA began with synthesizing graphene oxide (GO) nanosheets. Initially, 0.5 g of graphite powder was combined with 23 mL of 98% H2SO4 and agitated on ice. Next, 0.5 g of NaNO3 and 3 g of KMnO4 were added to the mixture, stirring for 3 h at 35 °C. After this, a solution of deionized water and 30% H2O2 was added. The mixture was filtered and washed with deionized HCl (pH 5). The GO nanosheets underwent sonication, and the remaining unexfoliated material was removed by centrifugation at 3500 rpm for 30 min.

To synthesize GQDs, GO powder was combined with 10 mL of dimethylformamide (DMF) and sonicated for 30 min. This step was followed by a solvothermal process at 200 °C for 5 h. Afterward, the GQD powder was filtered, freeze-dried, and dissolved in deionized water. In the next step, the carboxyl groups of HA were activated in an MES buffer (0.1 M) containing EDC/NHS (pH 5). After DA incubation, the activated HA was subjected to dialysis and lyophilization. Subsequently, the synthesized DA-HA was combined with GQD in Tris-buffered saline (TBS) and stirred for 24 h at 42 °C. DA links HA to GQD through the formation of amide bonds. Following this step, the mixture was purified through dialysis to produce the GQD-HA NCs. Finally, quinoline (10 mg) was dissolved in deionized water, mixed with the GQD-HA solution, and stirred for 24 h. Unbound quinoline was removed via dialysis, and the resulting dialyzed solution was subjected to lyophilization before being stored at 4 °C^9,23^.

### Characterization of GQD-HA-Qu NCs

As detailed elsewhere^24^, the hydrodynamic size and distribution of the GQD-HA-Qu NCs in an aqueous solution were investigated using dynamic light scattering (DLS). Furthermore, field emission scanning electron microscopy (FESEM), Transmission electron microscopy (TEM), Fourier transform infrared spectroscopy (FTIR), and X-ray diffraction (XRD) analysis were employed to assess the particle morphology and functional groups. The encapsulation efficiency (%) of quinoline in GQD-HA-Qu NCs was calculated and reported by the spectrophotometer absorption method and drawing a standard curve diagram^25^.

### Cytotoxicity assay

The toxic effects of GQD-HA-Qu NCs on various cancer cell lines, including MCF7, HT29, A2780, HeLa, and PANC-1, were examined using the MTT (3-[4,5-dimethylthiazol-2-yl]−2,5-diphenyl tetrazolium bromide) assay. The human dermal fibroblast (HDF) cell line was used as a control. The cells were seeded at a density of 1 × 10^4^cells per well in 96-well plates and incubated for 24 h. Subsequently, the cells were exposed to GQD-HA-Qu NCs at concentrations of 7.8, 15.6, 31.2, 62.5, 125, 250, and 500 µg/mL. Following a 48-hour incubation, 20 µL of MTT reagent (5 mg/mL) was added to each well, and the plates were incubated in the dark at 37 °C for 4 hours. After removing the medium, 200 µL of dimethyl sulfoxide (DMSO; Sigma) was added to facilitate the dissolution of the formazan crystals for one hour. Subsequently, the absorbance was measured at 570 nm using a microplate reader^25^.

### Annexin V-FITC/PI assay

Apoptosis was assessed using the Annexin V-FITC apoptosis staining detection kit (Abcam, ab14085) according to the manufacturer’s instructions. Following a 48-hour exposure of A2780 cells to GQD-HA-Qu NCs at concentrations of 10, 20, and 120 µg/mL, the cells were trypsinized and centrifuged. Subsequently, 1 × 10^5^ cells were suspended in 500 µL of binding buffer (1X) and incubated with 5 µL of annexin V-FITC and 5 µL of propidium iodide (PI) at 25 °C for 5 minutes in the dark. The cell suspension was centrifuged, and the cell pellet was resuspended in 400 µl of 1X binding buffer. Finally, the cell suspension was analyzed using flow cytometry to determine the percentage of early apoptotic cells (Annexin V+, PI-), late apoptotic cells (Annexin V+, PI+), and necrotic cells (Annexin V-, PI+). It must be noted that the 20 µg/mL concentration corresponds to the IC50 concentration, while the other two doses were chosen to induce cell death at around 25% and 75%, respectively.

### Real-time polymerase chain reaction (real time-PCR)

The expression levels of the p53, caspase 8, and caspase 9 genes were evaluated using real-time polymerase chain reaction (PCR). Briefly, RNA was extracted from A2780 cells using a QIAGEN total RNA extraction kit, and the extracted RNA was quantified and its quality assessed using a ThermoFisher Nanodrop ND-1000 spectrophotometer at 260 nm. The isolated mRNA was then transcribed into complementary DNA (cDNA) using Moloney murine leukemia virus reverse transcriptase (M-MLV RT; Promega). Subsequently, real-time PCR was conducted using a Corbett thermal cycler, specific primers (Table 1), and Bio-Rad SYBR Green Master Mix. The comparative cycle threshold (Ct) method was employed to normalize gene expression levels, utilizing glyceraldehyde-3-phosphate dehydrogenase (GAPDH) as a reference gene. The mean Ct values from triplicate measurements were used to calculate the expression level of the target gene using the 2^−ΔΔCt^ formula.

**Table 1:** Specific primer sequences for real-time PCR.

Primer	Sequence
P53	F: 5′-TCA GAT CCT AGC GTC GAG CCC -3′
	R: 5′-GGG TGT GGA ATC AAC CCA CAG -3′
Caspase 8	F 5′-GAAAAGCAAACCTCGGGGATAC-3′
	R 5′-CCAAGTGTGTTCCATTCCTGTC-3′
Caspase 9	F 5′- CCAGAGATTCGCAAACCAGAGG-3′
	R 5′- GAGCACCGACATCACCAAATCC-3′
GAPDH	F 5′- GCAGGGGGGAGCCAAAAGGGT-3′
	R 5′- TGGGTGCCAGTGATGGCATGG-3′

### Antioxidant activity of GQD-HA- Qu

The antioxidant capacity of GQD-HA-Qu NCs was evaluated by neutralizing ABTS and DPPH free radicals. Glutathione served as a control for the antioxidant assays. In the ABTS assay, a stock solution was prepared by combining equal volumes of 7 mM ABTS and 2.45 mM potassium persulfate, which was then incubated in the dark at 25 °C for 12 h. Subsequently, the solution was diluted with ethanol to an absorbance of 0.70 at 734 nm. Different concentrations of GQD-HA-Qu NCs were then introduced into the diluted ABTS solution in a 96-well plate, and the absorbance was measured after 6 min. For the DPPH assay, a 0.1 mM DPPH solution was prepared in methanol and incubated for 30 min in the dark. Subsequently, different concentrations of GQD-HA-Qu were introduced, and the absorbance was measured at 517 nm. The radical scavenging activity for both ABTS and DPPH assays was determined using the following formula for percentage inhibition: (control absorbance – sample absorbance) / control absorbance × 100.

### Statistical analysis

Statistical analysis was performed using SPSS software. The normality of the data was assessed using the Shapiro-Wilk test. A one-way analysis of variance (ANOVA) was conducted to compare means among multiple groups. Post hoc comparisons were conducted using Tukey’s test to identify specific differences between groups. A p-value of less than 0.05 was considered to indicate statistical significance. All data were presented as standard deviations (SD).

## Results

This study aimed to assess the influence of GQD-HA-Qu NCs on MCF-7, HT-29, A2780, HeLa, and PANC-1 cell lines, with a particular focus on their cytotoxicity and antioxidant capacity.

### DLS assay

**Fig. 1:**
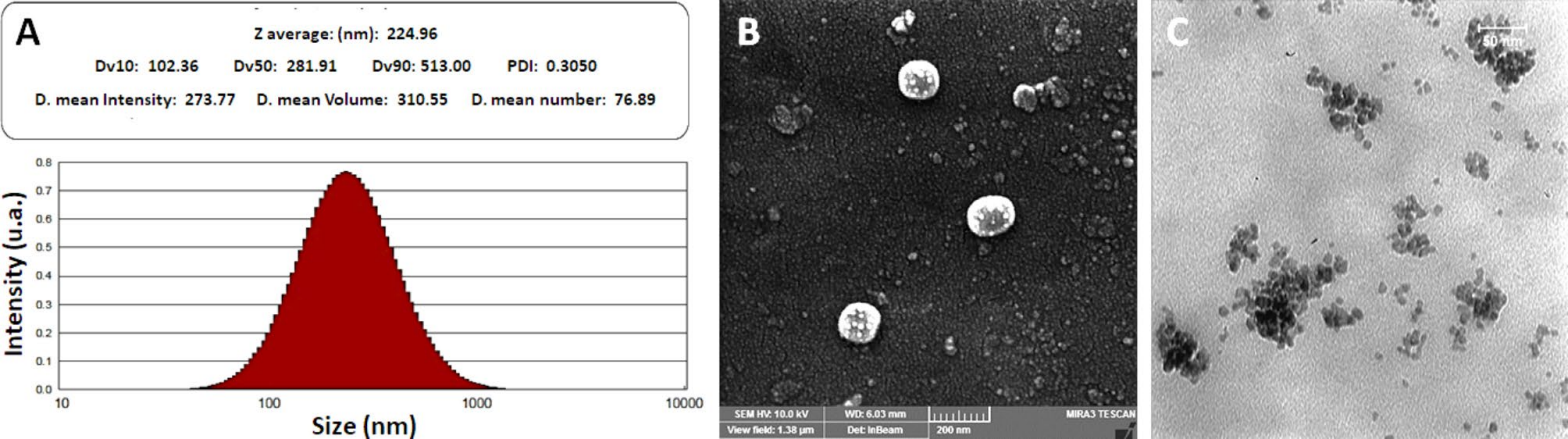
(**A**) The DLS assay for GQD-HA-Qu shows a Z-average particle size of 224.96 nm, a PDI of 0.3, and a mean number diameter of 76.89 nm, with a zeta potential of −43.38 mV. (**B**) The field emission scanning electron microscopy (FESEM) micrograph displays a dispersed population of spherical GQD-HA-Qu NCs with a narrow distribution, indicating successful conjugation and a stable colloidal system. (**C**) The transmission electron microscopy (TEM) micrograph of GQD reveals particles with spherical to irregular shapes, measuring under 10 nm in size, typical of a heterogeneous distribution of quantum dots.

As illustrated in Fig. 1A, the DLS analysis of GQD-HA-Qu NCs reveals a Z-average particle size of 224.96 nm, which falls within the generally accepted range for nanocarriers (1 nm to 1000 nm). The PDI of 0.3 indicates a relatively uniform size distribution, a critical factor for drug delivery in targeted therapy. The mean intensity diameter was 273.77 nm, while the mean volume diameter was 310.55 nm, confirming well-defined particle sizes. Additionally, the mean number diameter of 76.89 nm indicates a significant proportion of smaller particles, which may enhance cellular uptake and improve bioavailability. The negative zeta potential of −43.38 ± 19.64 mV indicates a substantial negative surface charge, contributing to the NCs’ suspension stability and preventing aggregation. These characteristics suggest that GQD-HA-Qu NCs possess favorable properties as nanocarriers, highlighting their potential for practical applications in drug delivery.

The FESEM (Fig. 1B) micrograph shows a dispersed population of spherical NCs consistent with the anticipated morphology of GQD-HA-Qu NCs. The nanoparticles exhibit a narrow size distribution, indicative of a controlled and homogeneous synthesis process. The scale bar confirms that the NCs fall within the nanometer range, consistent with the definition of NCs. The spherical morphology and uniform size distribution could enhance the effectiveness of DDS. The TEM (Fig. 1C) micrograph shows the size and morphology of GQD. The image reveals a spherical to irregular morphology and dimensions below 10 nm. The varied shapes and sizes of the particles indicate a heterogeneous distribution, characteristic of quantum dots.

### FTIR spectroscopy

**Fig. 2:**
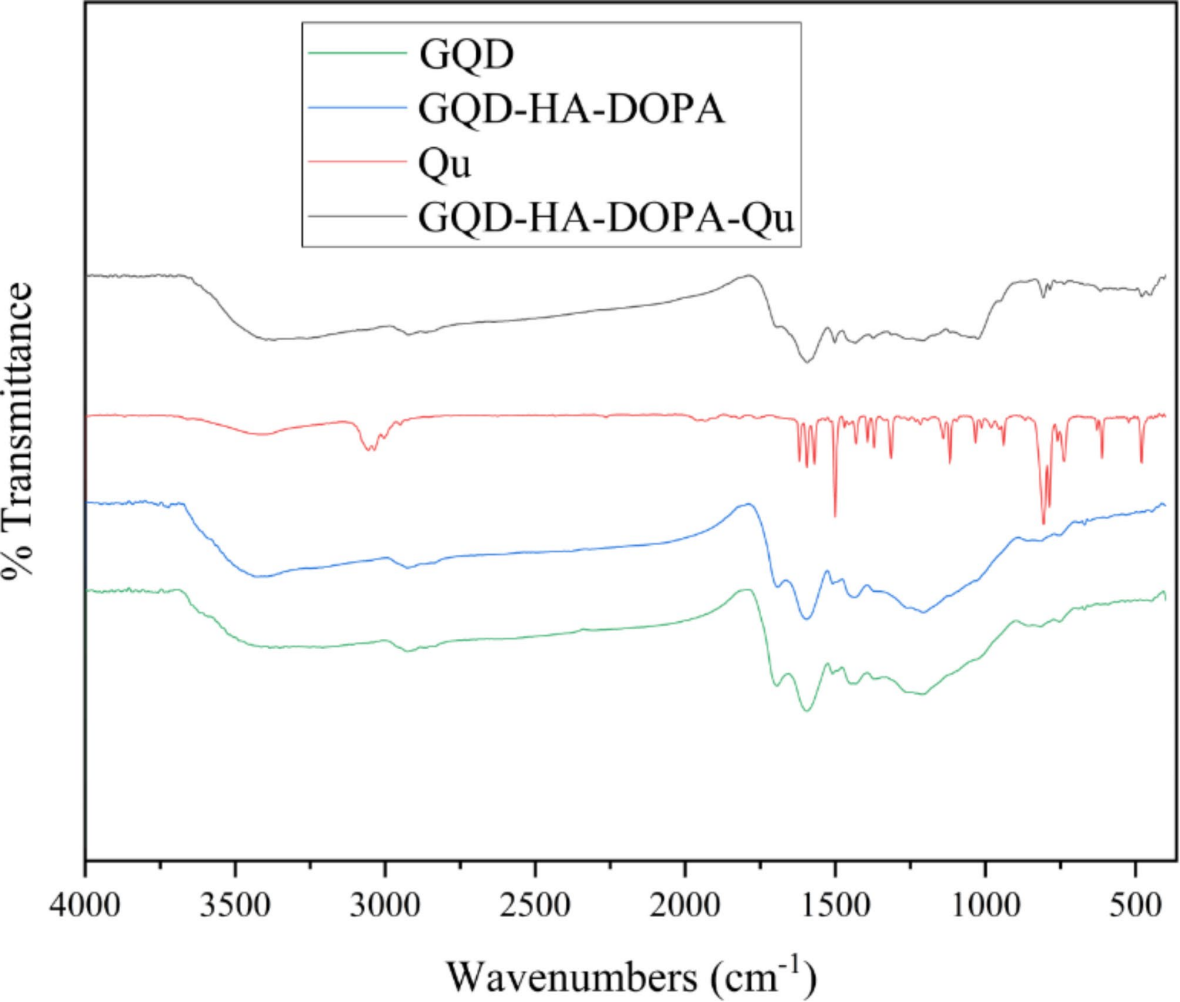
FTIR spectra of Quinoline, GQD, GQD-HA-DOPA, and GQD-HA-DOPA-Qu NCs. The analysis shows distinct absorption peaks for each compound, indicating their unique functional groups. Quinoline exhibits peaks for N-H stretching (3404.24 cm^−1^), aromatic C-H stretching (3056.66 cm^−1^ and 3036.04 cm^−1^), and C = C stretching (1619.89 cm^−1^ and 1595.29 cm^−1^). GQD displays broad peaks at 3382 cm^−1^ (hydroxyl groups) and a C = O peak at 1695 cm^−1^. The GQD-HA-DOPA spectrum indicates successful incorporation of hyaluronic acid and DOPA, while GQD-HA-DOPA-Qu shows peaks for hydroxyl, amine, and carbonyl groups, reflecting quinoline integration.

Figure 2 shows the FTIR spectra of quinoline, GQD, GQD-HA-DA, and GQD-HA-DA-Qu NCs. The FTIR analysis of quinoline shows several absorption peaks that indicate various functional groups, including a broad peak at 3404.24 cm^−1^ for N-H stretching, peaks around 3056.66 cm^−1^ and 3036.04 cm^−1^ for C-H stretching in aromatic compounds, and peaks at 1619.89 cm^−1^ and 1595.29 cm^−1^ corresponding to C = C stretching. Additional peaks at 1500.88 cm^−1^ and 1431.44 cm^−1^ suggest C-N vibrations, while 1140.52 cm^−1^ and 1118.24 cm^−1^ indicate C-O stretching.

The FTIR spectrum of GQD displays a broad peak around 3382 cm^−1^, indicating the presence of hydroxyl (-OH) groups, suggesting surface functionalization. Peaks at 2926 cm^−1^ and 2864 cm^−1^ correspond to C-H stretching vibrations of aliphatic hydrocarbons, while a significant peak at 1695 cm^−1^ relates to C = O stretching, likely from carbonyl or carboxyl groups. Additional peaks at 1429 cm^−1^ and 1262 cm^−1^ are attributed to C-H bending and C-O stretching vibrations, respectively.

The GQD-HA-DOPA FTIR spectrum reveals a broad peak at 3382 cm^−1^, which signifies hydroxyl (-OH) groups, reflecting hyaluronic acid’s hydrophilicity. Peaks at 2926 cm^−1^ and 2864 cm^−1^ correspond to C-H stretching vibrations typical of aliphatic hydrocarbons, while a significant peak at 1695 cm^−1^ indicates C = O stretching from carbonyl or carboxyl groups. Peaks show aromatic C = C bending at 1595 cm^−1^ and 1507 cm^−1^, and C-H bending is reflected in peaks at 1429 cm^−1^ and 1372 cm^−1^. Additional peaks at 1262 cm^−1^ and 1212 cm^−1^ suggest C-O stretching, with 857 cm^−1^, 808 cm^−1^, and 755 cm^−1^ indicative of out-of-plane C-H bending. Overall, the spectrum confirms the successful functionalization of HA and DOPA with GQD.

In addition, the FTIR spectrum of GQD-HA-DOPA-Qu shows a broad peak at 3370.10 cm^−1^ for O-H and N-H stretching, indicating hydroxyl and amine groups from HA and DOPA. Additional N-H stretching is at 3256.30 cm^−1^. The 2922.73 cm^−1^ and 2859.93 cm^−1^ peaks also correspond to C-H stretching vibrations, typical for aliphatic and aromatic structures. The peak at 1695.35 cm^−1^ indicates C = O stretching, likely from carboxylic acid or amide groups. Peaks at 1593.83 cm^−1^ and 1501.97 cm^−1^ reflect C = C stretching in quinoline, while peaks at 1208.49 cm^−1^ and 1049.72 cm^−1^ suggest C-O stretching from ester or ether linkages in HA. Overall, this FTIR analysis highlights the complex interplay of functional groups in the hybrid material, emphasizing its potential applications in biomedicine and nanotechnology.

### XRD analysis

**Fig. 3:**
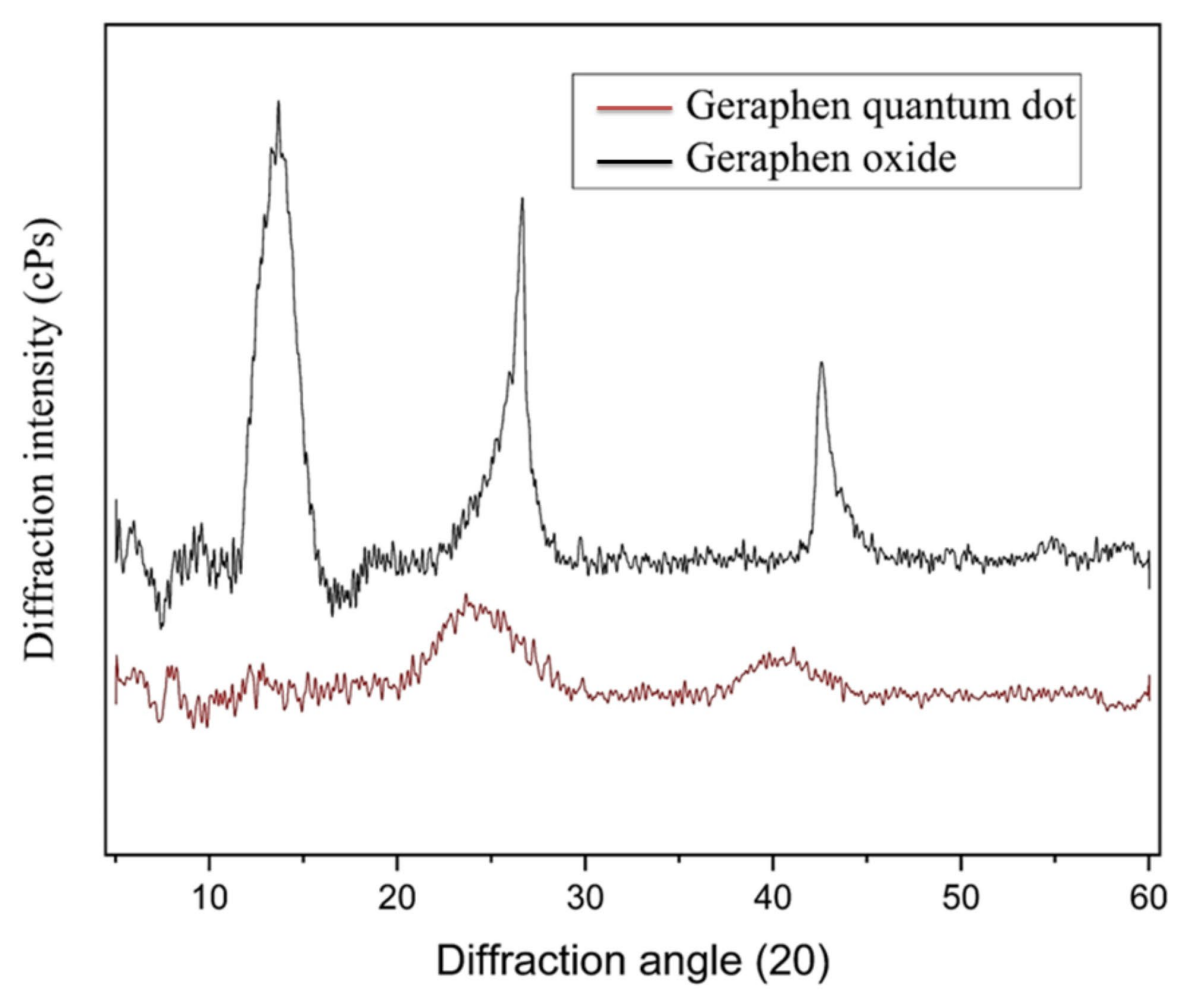
The X-ray diffraction (XRD) patterns of graphene oxide (GO) and GQDs. XRD analysis of graphene oxide and GQDs reveals distinct structural properties. Graphene oxide shows prominent peaks around 10–15° (2θ) associated with the (001) plane, indicating increased interlayer spacing and some crystallinity, alongside a peak near 25–30° (2θ) linked to residual graphitization. GQDs have broad peaks in the same 10–15° range, reflecting their amorphous nature, plus a significant peak at 25–30° due to partial graphitization. Smaller peaks at higher angles suggest complex carbon structures or impurities. Overall, the results confirm the successful synthesis of both materials, emphasizing the differences in crystallinity.

As shown in Fig. 3, the XRD pattern of graphene oxide features distinct peaks, with a prominent peak around 10–15° (2θ) indicating the (001) plane, reflecting increased interlayer spacing due to oxygen functional groups and suggesting some crystallinity. A 25–30° (2θ) peak may correspond to the (002) plane of reduced graphene oxide or residual graphite, indicating some graphitization. Smaller peaks at higher angles (40–60°) could represent other carbon structures or impurities. Overall, the defined peaks and low background noise confirm the successful synthesis of graphene oxide, though the lower peak intensity suggests decreased crystallinity and long-range order.

Similarly, the XRD pattern of GQDs shows broad, low-intensity peaks centered around 10–15° (2θ), indicating the interlayer spacing characteristic of graphene oxide (GO) and reflecting their amorphous nature. A significant peak near 25–30° (2θ) suggests some degree of graphitization, while smaller peaks at higher angles point to a complex structure. Overall, the XRD results confirm the amorphous nature of GQDs, the presence of functional groups (though potentially less extensive than in graphene oxide), and a reduction in crystallinity compared to pristine graphene, consistent with their synthesis through controlled oxidation or functionalization.

### Encapsulation efficacy

**Fig. 4:**
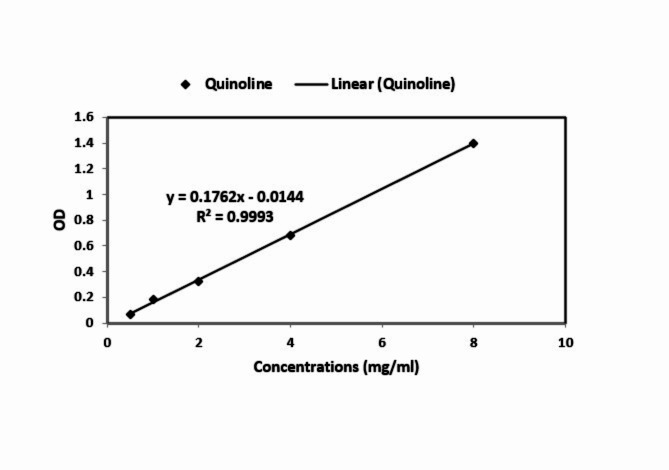
Standard curve obtained from different concentrations of quinoline at a wavelength of 289 nm.

The spectrophotometric method assessed the encapsulation efficiency at a wavelength of 289 nm (Fig. 4). The measured 90.32% encapsulation efficiency indicates the successful incorporation of a significant amount of quinoline into the GQD-HA NCs. These results confirm the capability of the GQD-HA NCs to conjugate with quinoline effectively.

### Cytotoxicity assay

**Fig. 5:**
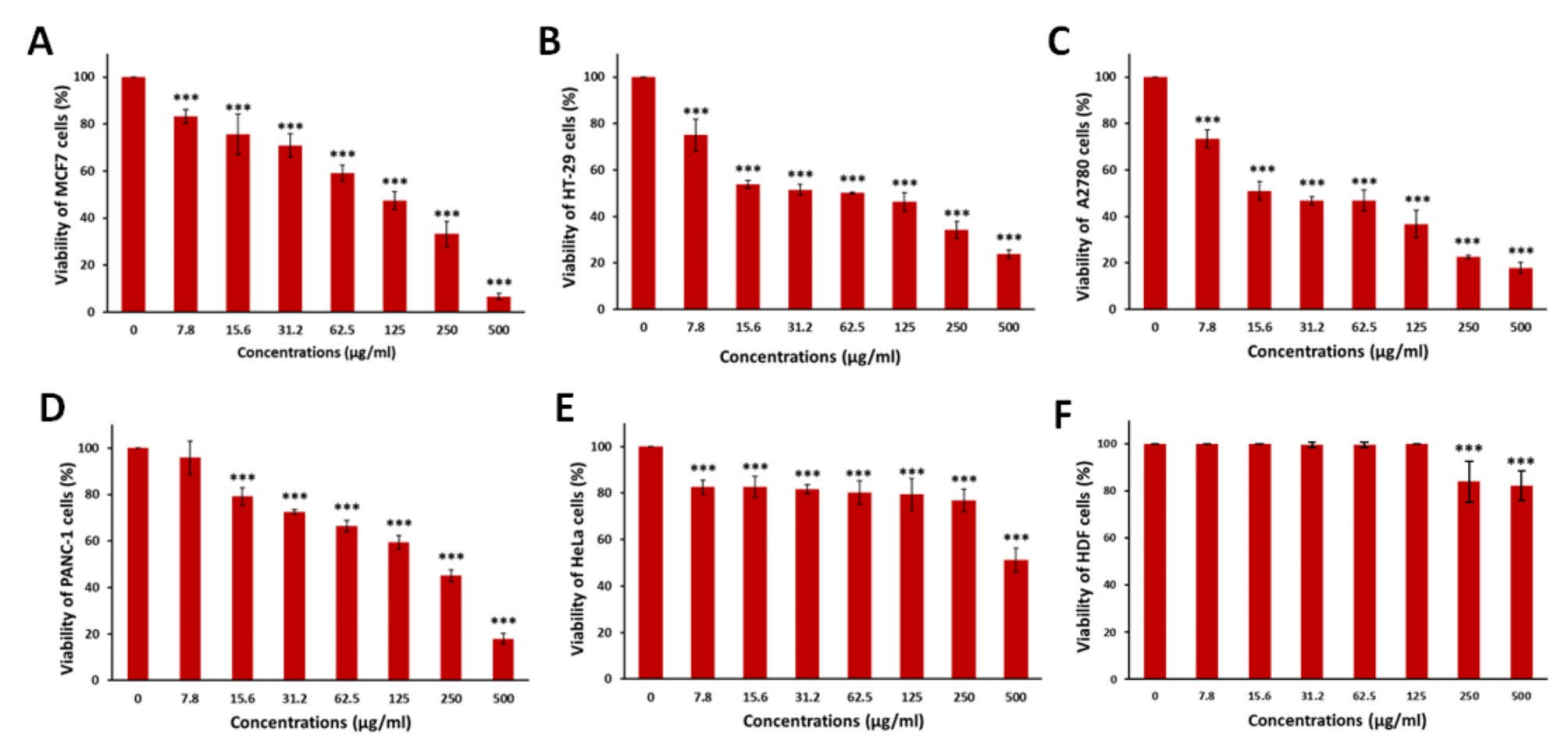
Cytotoxic effects of GQD-HA-Qu NCs on various cancer cell lines. The MTT assay results show a concentration-dependent reduction in cell viability for MCF-7 (**A**), HT-29 (**B**), A2780 (**C**), PANC-1 (**D**), and HeLa (**E**) cells. A2780 cells exhibited the highest sensitivity, followed by HT-29, while MCF-7 cells demonstrated greater sensitivity than HT-29 at 250 µg/mL and 50 µg/mL and more sensitivity than A2780 at 500 µg/mL. In contrast, HeLa cells showed the highest resistance, maintaining significant viability, and PANC-1 cells displayed minimal response to lower concentrations, indicating a need for higher doses for an effective response. The data is presented as mean ± standard deviation (SD), and statistical significance is denoted as *** *P* < 0.001.

The MTT assay revealed the cytotoxic effects of varying concentrations of GQD-HA-Qu NCs on MCF-7, HT-29, A2780, PANC-1, and HeLa cancer cell lines (Fig. 5). According to the results, the IC50 values for the MCF-7, HT-29, A2780, PANC-1, and HeLa cell lines were 112, 62, 20, 208, and > 500, respectively. The findings demonstrate a reduction in cell viability in all cell lines as the concentration of GQD-HA-Qu NCs increased. Overall, A2780 and HT-29 cells exhibited the highest sensitivity, but MCF-7 cells showed greater sensitivity at 500 µg/mL. In contrast, HeLa cells exhibited the highest resistance, maintaining relatively high viability. PANC-1 cells also demonstrated notable resistance, requiring higher concentrations to elicit a measurable response. This differential response among cell lines underscores the potential for GQD-HA-Qu NCs to selectively target specific cancer types, which could benefit future therapeutic applications.

### Annexin V-FITC/PI assay

**Fig. 6:**
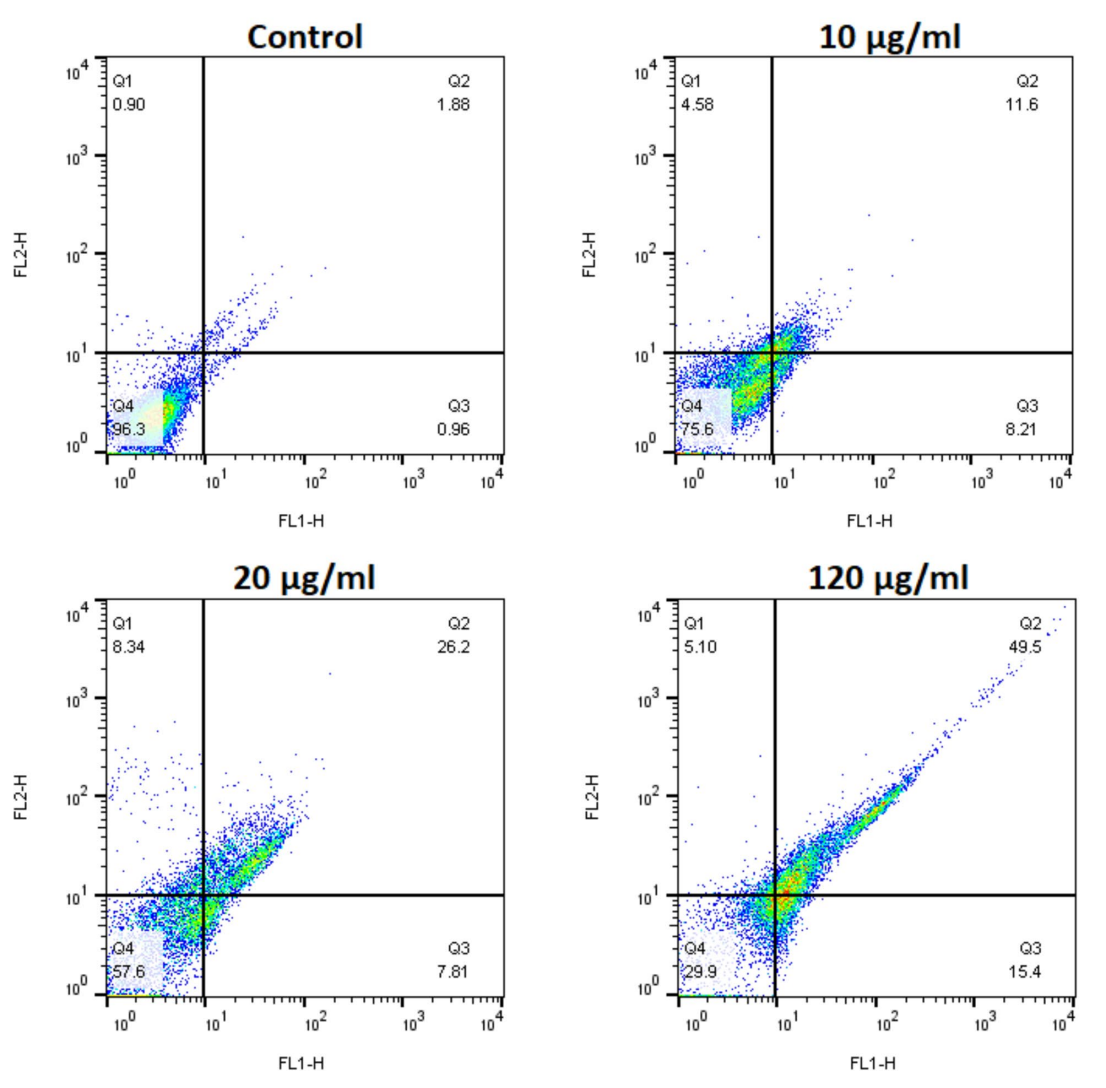
Apoptosis induction by GQD-HA-Qu NCs. Flow cytometry results show a dose-dependent increase in apoptotic cells with GQD-HA-Qu NCs concentration, from 10 µg/mL (early: 8.21%, late: 11.6%) to 120 µg/mL (early: 15.4%, late: 49.5%), indicating significant induction of apoptosis as cell viability decreases.

The flow cytometry results demonstrate an apparent shift in cell distribution with increased GQD-HA-Qu NCs concentrations from 10 to 120 µg/ml. This shift is accompanied by a growing proportion of double-positive cells for Annexin V and PI, which indicates late apoptosis or necrosis. As illustrated in Fig. 6, Untreated cells exhibited high viability (96.3%), while treatment with GQD-HA-Qu NCs resulted in increased early (8.21%) and late (11.6%) apoptotic cells at 10 µg/mL. At 20 µg/mL, the percentage of late apoptotic cells increased to 26.2%. At 120 µg/mL, there was a significant increase in both early (15.4%) and late (49.5%) apoptotic cells, indicating a substantial induction of apoptosis. These findings imply a dose-dependent effect of the GQD-HA-Qu NCs treatment on apoptosis induction in cancer cells.

### Apoptotic gene expression

**Fig. 7:**
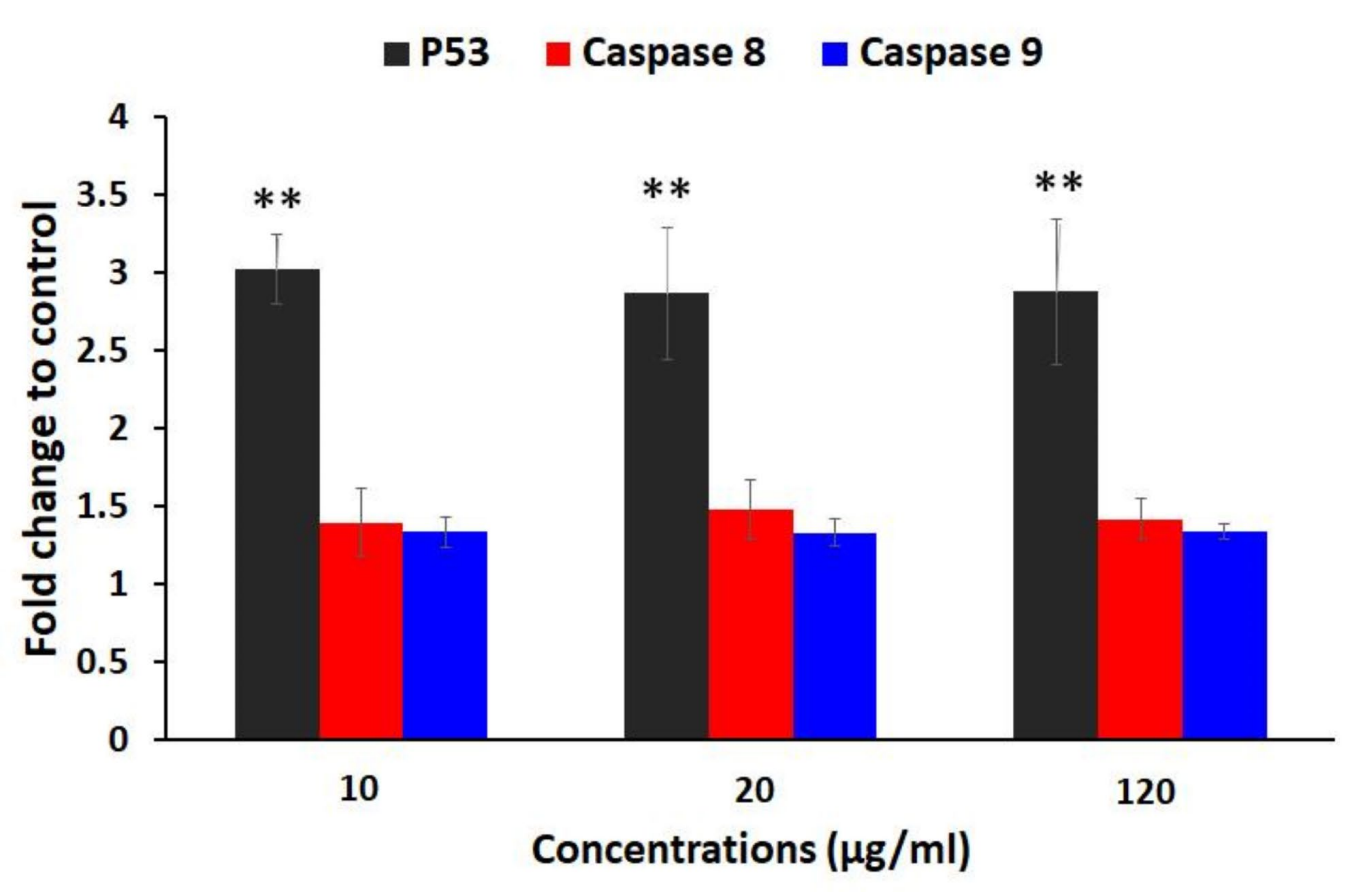
Real-time PCR assay. The mRNA expression levels of p53, caspase 8, and caspase 9 in cancer cells treated with GQD-HA-Qu NCs at 10, 20, and 120 µg/mL showed significant upregulation of p53. In contrast, caspase 8 and 9 levels remained unchanged, indicating a primary effect on the p53 pathway in regulating apoptosis and cell cycle arrest. The data is presented as mean ± standard deviation (SD), and statistical significance is denoted as ** *P* < 0.01.

Figure 7 depicts mRNA expression levels of the p53, caspase 8, and caspase 9 genes in cancer cells treated with GQD-HA-Qu NCs at 10, 20, and 120 µg/mL concentrations. Results show significant upregulation of p53 at all concentrations compared to untreated cells (*P* < 0.05). However, no notable alterations were observed in caspase 8 and 9 expressions compared to controls. These findings indicate that GQD-HA-Qu NCs primarily affect the p53 pathway, suggesting robust activation of this tumor suppressor gene, which is crucial for regulating apoptosis and cell cycle arrest.

### Antioxidant capacity assay

**Fig. 8:**
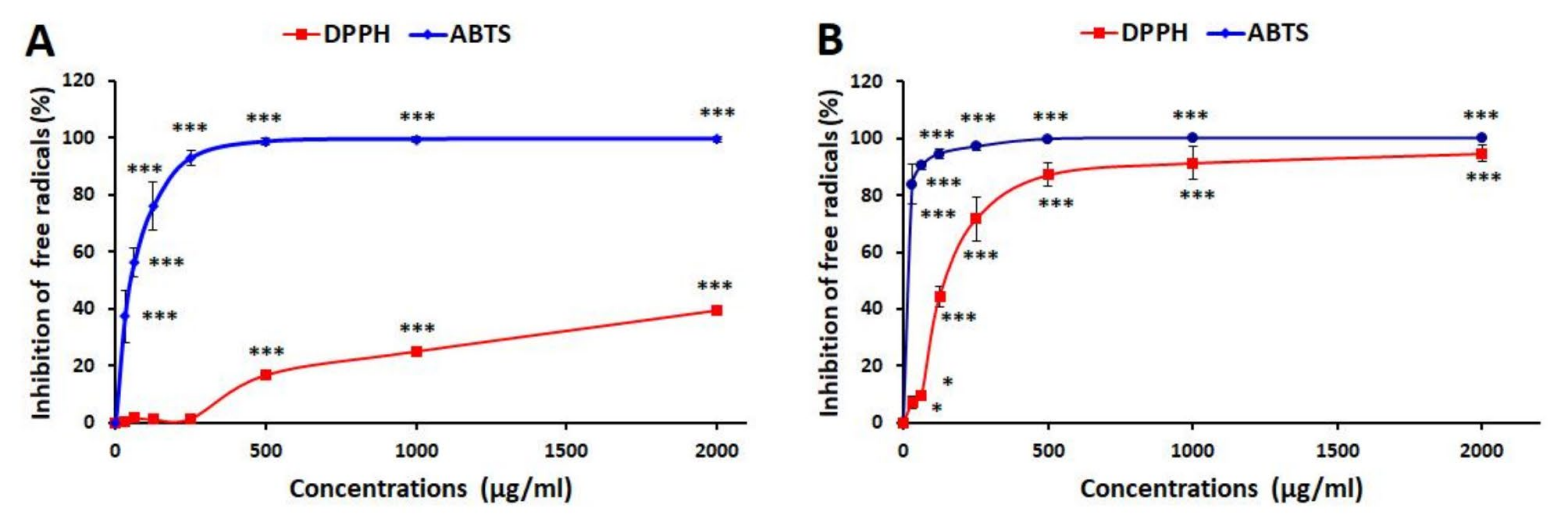
Antioxidant activity of GQD-HA-Qu NCs (**A**) and glutathione (**B**). The data is presented as mean ± standard deviation (SD), and statistical significance is denoted as *** *P* < 0.001.

The antioxidant capacity of GQD-HA-Qu NCs was assessed by measuring their ability to neutralize ABTS and DPPH free radicals (Fig. 8A). According to the results, the GQD-HA-Qu NCs exhibit an IC50 value of 51.9 µg/mL for ABTS inhibition and > 2000 µg/mL for DPPH. At 500 µg/mL and above concentrations, GQD-HA-Qu NCs exhibited significant inhibitory effects on DPPH free radicals. Furthermore, these particles demonstrated considerable capacity to scavenge ABTS free radicals across all tested concentrations, achieving nearly complete inhibition at around 2000 µg/ml. On the other hand, glutathione shows IC50 values of 18.63 µg/mL for ABTS inhibition and 150.43 µg/mL for DPPH (Fig. 8B). Although GQD demonstrated good antioxidant properties, its antioxidant capacity was lower than glutathione’s.

## Discussion

The cancer mortality rate is steadily increasing, highlighting the urgent need for advancements in cancer treatment a crucial and ongoing research priority^26^. However, current cancer drug therapies face limitations regarding tumor specificity, adverse effects, and resistance^27^. In recent years, there has been a notable shift in cancer treatment approaches toward target therapy, which aims to eliminate cancer cells while sparing normal cells^28^. To address this approach, using nitrogen heterocyclic compounds, such as quinoline, in conjunction with NCs as nanocarriers can potentially enhance the efficacy of anti-cancer drug development. In this context, the present study examined the impact of quinoline-loaded GQD-HA NCs on a range of cancer cell lines, including MCF7, HT29, A2780, HeLa, and PANC-1. The study’s objectives were to assess the efficacy of quinoline encapsulation, evaluate the loaded GQD-HA NCs’ cytotoxicity, and determine the NCs’ antioxidant capacity.

Our results indicate a Z-average particle size of 224.96 nm with a polydispersity index of 0.3, signifying uniformity and stability for drug delivery. A robust negative zeta potential of −43.38 ± 19.64 mV contributes to the stability of the suspension and prevents aggregation. Further, FESEM imaging corroborates the spherical morphology of the nanoparticles, while FTIR confirms the presence of functional groups. Cytotoxicity assays reveal that A2780 cells are particularly susceptible to GQD-HA-Qu NCs, whereas HeLa and PANC-1 cells demonstrate considerable resistance, indicating potential for selective targeting. Flow cytometry results show a dose-dependent increase in the proportion of apoptotic cells, especially at higher concentrations. Concurrently, gene expression analysis evinces a considerable elevation in p53 gene expression, underscoring its function in apoptosis regulation. Furthermore, the antioxidant assay demonstrated the significant effectiveness of GQD-HA-Qu NCs in scavenging DPPH and ABTS free radicals, particularly at concentrations of 500 µg/mL and above. These findings collectively indicate that GQA-HD-Qu NCs possess commendable properties that make them suitable for targeted cancer therapies, exhibiting notable cytotoxic and antioxidant activities.

GQD and GQD-HA nanocarriers for targeted cancer therapy have shown promising results^9,14^. Evidence suggests that combining bioactive compounds with these nanocarriers enhances their effectiveness through some mechanisms, including improved solubility, precise delivery, controlled release, facilitated cellular uptake, and protection from degradation^12^. For example, Vahedi et al. (2021) optimized GQD for cancer treatment by conjugating it to HA. The resulting GQD-HA nanocarriers exhibited a loading efficiency of 98.02% for curcumin. Furthermore, GQD-HA-curcumin notably reduced HeLa cell viability while maintaining biocompatibility with L929 cells^9^. In another study conducted by Lin et al. in 2024, a dual DDS was developed by combining positively charged GQDs with modified polyethyleneimine (PEI) and negatively charged HA-containing pyrenebutyric acid. The resulting particles, GPI and HANPs, showed high drug-loading efficiency and hydrogel-like properties. The simultaneous delivery of both drugs resulted in an enhanced therapeutic effect. The successful synthesis was confirmed, and the system’s efficacy was evaluated using MTT assays on HCT116 cancer cells and in vivo experiments with mouse xenografts. Their results demonstrated that the dual delivery system, HANPs (TAK)/GPI (DOX), markedly suppressed cancer cell proliferation, thereby establishing its potential as a promising candidate for novel cancer therapies^3^. Ghanbari et al. (2024) developed a controlled-release DDS using GQDs to enhance tamoxifen (TMX) efficacy against breast cancer. The GQDs were conjugated with folic acid (FA) as a targeting agent, and TMX was bound non-covalently. The GQD-FA-TMX NCs exhibited pH-sensitive release profiles. The MTT assays showed that the GQD-FA-TMX NCs had significantly higher cytotoxicity against MCF-7 cells than GQDs and free TMX^7^.

Likewise, quinoline is a heterocyclic aromatic compound and a pharmacophore characterized by its double-ring structure comprising a benzene ring and a pyridine ring^29^. Biologically, quinoline and its derivatives exhibit a range of therapeutic features, including antimalarial^30^, antibacterial, antifungal^31^, antiviral^32^, analgesic^33^, and anticonvulsant^34^properties. Moreover, they possess significant anti-inflammatory^35^and antioxidant properties^36^, making them effective in managing inflammatory disorders. Besides, the quinoline scaffold plays a vital role in developing anti-cancer drugs, and its derivatives have been screened for their anti-cancer potential^37^. It has been demonstrated that quinoline derivatives induce apoptosis in cancer cells^38^. Additionally, they inhibit cell proliferation by inducing cell cycle arrest^39^, inhibiting angiogenesis^40^, disrupting cell migration^41^, and modulating nuclear receptor responsiveness^42^, thereby slowing tumor growth. The potential of quinoline in cancer treatment is enhanced by its interference with key signaling pathways^42^.

Several studies have investigated the anti-cancer effects of quinoline, highlighting its efficacy against various cancer types and the underlying mechanisms involved. The anti-cancer capacity of quinoline and its derivatives is attributed to several mechanisms, including apoptosis induction, tyrosine kinase inhibition, cell cycle arrest, antimitotic tubulin polymerization, and angiogenesis inhibition^43^. Quinoline induces programmed cell death by activating various apoptotic pathways. It can modulate the expression of essential regulatory proteins that regulate the equilibrium between pro-apoptotic and anti-apoptotic signals within the cell. For instance, quinoline derivatives can upregulate pro-apoptotic proteins such as Bax and Bak, promoting mitochondrial outer membrane permeabilization and releasing cytochrome c into the cytosol. This process activates caspases, a family of proteases that play a crucial role in executing apoptosis^44,45^.

Conversely, quinoline and its derivatives can downregulate anti-apoptotic proteins, such as Bcl-2 and Bcl-xL, which typically inhibit cell death. By disrupting this balance, quinolines shift the scales in favor of apoptosis, particularly in cancer cells that often evade programmed cell death through overexpression of anti-apoptotic factors^46,47^. Moreover, these compounds may activate extrinsic apoptotic pathways by influencing death receptor signaling, further enhancing their ability to induce cell death in malignancies. Additionally, quinoline derivatives have been identified as potent inhibitors of various kinases, including the epidermal growth factor receptor (EGFR)^48,49^and the vascular endothelial growth factor receptor (VEGFR)^48^. These receptors are critical in regulating cellular signaling pathways that control cell proliferation, differentiation, and survival. Eliminating EGFR by quinolines effectively blocks the downstream signaling cascades that promote tumor growth and metastasis. Concurrently, the inhibition of VEGFR disrupts angiogenesis and the formation of new blood vessels that supply nutrients and oxygen to tumors^50^. Angiogenesis is a vital process for tumor growth and metastasis^51^. By targeting angiogenesis signaling pathways, quinoline derivatives can effectively disrupt the communication between tumor cells and endothelial cells, thereby preventing the formation of new blood vessels that supply nutrients and oxygen to tumors. This inhibition deprives the cancer cells of essential resources and diminishes their metastasizing capacity. This dual action not only hampers cancer cell proliferation but also enhances the sensitivity of tumors to other therapeutic agents. Selectively targeting these kinases by quinoline derivatives presents a promising approach for developing novel anti-cancer therapies, particularly for overcoming resistance to existing treatments.

Additionally, some quinoline derivatives have demonstrated the capacity to induce cell cycle arrest at the G2 and S phases, thereby preventing cancer cells from dividing and proliferating^52^. By targeting critical regulatory proteins involved in the cell cycle, these compounds disrupt the normal progression of the cell cycle. To illustrate, during the G2 phase, quinoline derivatives can inhibit key checkpoints responsible for ensuring the integrity of the DNA prior to mitosis, resulting in the accumulation of cells that cannot progress to the division phase. In the S phase, these derivatives may disrupt the synthesis or repair of DNA, further hindering the progression of the cell cycle^53,54^.This targeted intervention halts the proliferation of cancer cells and increases the likelihood of apoptosis in cells with damaged DNA.

Some quinoline derivatives interact directly with DNA, leading to structural alterations inhibit replication and transcription^54^. These compounds can intercalate between DNA base pairs or bind to the DNA helix, causing distortions that impede the normal unwinding and separation of strands required for replication. This interference halts the synthesis of new DNA. It disrupts the transcription process, essential for producing messenger RNA (mRNA) and translating proteins necessary for cell survival and proliferation^55^. The resulting accumulation of unrepaired DNA damage triggers cellular stress responses, often leading to apoptosis in cancer cells that are more vulnerable due to their rapid growth and division^56^. Some quinoline derivatives are topoisomerase inhibitors, which are crucial enzymes for DNA replication and transcription^55^. The inhibition of these enzymes can result in DNA damage and apoptosis in cancer cells.

Quinolines’ capacity to selectively target malignant cells while sparing normal cells enhances their therapeutic potential. Further research into the specific binding mechanisms and structural characteristics of these quinoline-DNA interactions can facilitate the development of new anti-cancer agents. Additionally, many quinoline derivatives have been identified as potent inhibitors of tubulin polymerization, a process essential for forming microtubules during cell division. By interfering with this assembly, these compounds disrupt the normal dynamics of the mitotic spindle, leading to cell cycle arrest, particularly during the mitotic phase^57^. This disruption arrests the progression of cancer cells through the cell cycle and initiates a cascade of cellular events that ultimately result in apoptosis. The ability of quinoline derivatives to selectively target cancerous cells while sparing normal cells highlights their potential as effective therapeutics in cancer treatment. This evidence emphasizes the necessity for further research into their mechanisms of action and potential clinical applications^58^.

## Conclusion

In conclusion, this study demonstrates that GQD-HA-Qu NCs are promising DDS, characterized by favorable encapsulation efficacy, cytotoxicity against various cancer cell lines, and significant antioxidant capacity. The DLS and FESEM analyses confirm their appropriate size and morphology for targeted therapy. Additionally, the cytotoxicity assays reveal a differential sensitivity among cancer cell lines, highlighting the potential for selective targeting. The findings highlight the activation of the p53 pathway, suggesting that GQD-HA-Qu NCs can effectively induce apoptosis in cancer cells. These results support the potential application of GQD-HA-Qu NCs in cancer treatment. However, this study has several limitations, including its focus on in vitro cell lines, which may not accurately reflect in vivo interactions and microenvironments. Additionally, the long-term effects and potential toxicity of GQD-HA-Qu NCs were not explored, and the differential sensitivity among cancer cell lines was not examined in detail. Future research should optimize the formulation for specific cancer types, evaluate the therapeutic effects over extended periods, and investigate combinations with other treatment modalities to enhance the overall anti-cancer effectiveness while ensuring biocompatibility and minimizing side effects.

## Data Availability

Data will be made available on request of Mozhgan Soltani.
